# The chronic disease helplessness survey: developing and validating a better measure of helplessness for chronic conditions

**DOI:** 10.1097/PR9.0000000000000991

**Published:** 2022-03-14

**Authors:** Lindsey R. Yessick, Tim V. Salomons

**Affiliations:** a Departments of Psychology, Queen's University, Kingston, ON, Canada; b Centre for Neuroscience Studies, Queen's University, Kingston, ON, Canada

**Keywords:** Learned helplessness, Perceived control, Chronic pain, Questionnaire

## Abstract

**Introduction:**

Learned helplessness develops with prolonged exposure to uncontrollable stressors and is therefore germane to individuals living with pain or other poorly controlled chronic diseases. This study has developed a helplessness scale for chronic conditions distinct from previous scales that blur the conceptualization of control constructs. Extant measures commonly examine controllability, not the three pillars of helplessness identified by Maier and Seligman (1976): cognitive, emotional, and motivational/motor deficits.

**Methods:**

Individuals who self-report a chronic pain condition (N = 350) responded to a Chronic Disease Helplessness Survey (CDHS) constructed to capture cognitive, motivational/motor, and emotion deficits. Exploratory factor analysis (EFA; N = 200) and confirmatory factor analysis (CFA; N = 150) were performed. The CDHS was assessed for convergent and discriminant validity.

**Results:**

A three-factor solution corresponding to cognitive, emotional, and motivational/motor factors was identified by EFA. The solution exhibited sufficient model fit and each factor had a high degree of internal consistency. The CDHS was significantly associated with greater pain intensity and interference, PCS helplessness, lower perceived pain control, and lower general self-efficacy. Individuals with diabetes generally experience greater control strategies over daily symptoms (e.g., diet, oral medications, and insulin) than patients with chronic pain and in this study displayed significantly lower CDHS scores compared to individuals with chronic pain, demonstrating discriminant validity.

**Conclusions:**

This study provides preliminary evidence that the three-factor CDHS is a psychometrically sound measure of helplessness in individuals with chronic pain.

## Abstract 2

Supplemental Digital Content is Available in the Text.

## 1. Introduction

Over 5 decades ago, learned helplessness (LH) was originally observed and described as a failure to learn to escape an avoidable aversive stimulus after an organism has been exposed to unavoidable aversive stimuli.^23,29^ Constellations of observable behaviors that result from LH were described in the original proposal of the helplessness hypothesis, in which Maier and Seligman^19^ indicated that the learned disconnection of behavioral actions and outcomes produces motivation/motor (one's ability to initiate and maintain physical motivation or energy), cognitive (one's ability to learn new ways to escape uncontrollable stress), and the emotion (one's ability to manage their emotional responses to uncontrollable stress) deficits. Because LH develops with exposure to repeated stressors and no perceived escape strategy, LH is highly germane to chronic disease populations who may be exposed to an uncontrollable health stressor daily. Maier and Seligman's^19^ assertion that repeated exposure to uncontrollable stress results in a generalized failure to learn new contingencies between responses and outcomes results in the prediction that the degree to which a person with, for instance, chronic pain perceives their pain as uncontrollable will determine the degree to which they are able to learn new ways of dealing with their pain and the degree to which they generalize their lack of perceived control to life stressors other than pain. Reduced motivation and lack of belief that life stress can be controlled has been demonstrated to contribute to the maintenance of suffering in chronic disease, such as in the case of chronic pain.^12,14,16,17^

Extant measures of controllability and helplessness do not capture these constellations of LH behaviors (motivational, cognitive, and emotional effects), which are the frequent but not inevitable result of prolonged exposure to uncontrollable stress. There are individual differences in the likelihood of becoming helpless given the same level of exposure to uncontrollable stress, emphasizing the need for proper measurement of the helplessness-related behavioral consequences. The examination of control has been confused by a multitude of measures of correlated but separate constructs related to control beliefs (eg, locus of control, self-efficacy, mastery, helplessness, etc.).^31,36^ Although some extant self-report measures used in chronic pain populations putatively measure “helplessness,” these largely reflect the degree to which an individual believes they lack control over pain, rather than the behavioral and emotional sequelae. For example, a typical item on the helplessness subscale of the Pain Catastrophizing Scale is “There's nothing I can do to reduce the intensity of the pain.”^32^ Similar to other items in the subscale, this item assesses the individual's current assessment of the controllability of their pain, rather than assessing cognitive, motivational, or emotional deficits considered definitional of helplessness by Maier and Seligman.^19^

This study aimed to develop and validate a helplessness scale for chronic conditions both theoretically and methodologically distinct from previous scales that blur the conceptualization of control constructs and do not capture the domains of helplessness previously defined by Maier and Seligman.^19^ The development of such a tool would not only align clinical findings with a rich behavioral and biological literature but would more directly reflect the psychological impact of perceived lack of control. Learned helplessness is a common but not inevitable result of perceived lack of control. As such, a direct measure of the cognitive, motivational, and emotional dimensions of helplessness would help clinicians identify those individuals for whom controllability beliefs are critical to clinical presentation.

Because LH develops with exposure to repeated aversive stimuli and no perceived viable coping or control strategy, LH is highly relevant to chronic pain populations who are continuously exposed to aversive, uncontrollable pain. Therefore, this study was performed in individuals with chronic pain. We hypothesized that high Chronic Disease Helplessness Survey (CDHS) helplessness scores would be associated with more self-reported pain and would be associated, but not redundant, with interrelated pain control measures. As stated earlier, LH has been shown to result in motivational and motor deficits such as escape-related motor responses^1,10,23,29,40^, decreased aggressive and competitive behavior,^5,24^ and reductions in spontaneous motor behavior.^38^ For this reason, we predicted that LH would be associated with greater feelings of fatigue. Last, because individuals with diabetes titrate and monitor blood sugar levels daily using diet, oral medications, and insulin, we hypothesized that individuals with diabetes would perceive greater volitional control over their symptoms and would therefore be less likely to exhibit helplessness than individuals with chronic pain, who often perceive few viable options for symptom management. Therefore, we expect to discriminate between the amount of helplessness experienced by individuals with diabetes in comparison with those with chronic pain.

## 2. Method

### 2.1. Participants

Participants with chronic pain were recruited using Mechanical Turk. To be eligible, participants were required to have a 95% hit approval rate on MTurk, be aged at least18 years, reside in the United States, and self-report a chronic pain condition. Three hundred fifty individuals were included in analyses after screening (see Procedures for further screening methods). The first 200 eligible participants who responded to the survey were included to perform exploratory factor analysis and the final 150 for confirmatory factor analysis. Further sociodemographic data are listed in Table 1.

**Table 1:** Sociodemographic information.

	Chronic pain (N = 350)	Diabetes (N = 36)	*P*
Age (M [SD])	41.9 (13.2)	42.0 (15.7)	0.964
Biological sex (% [*n*])			0.806
Female	60.3 (211)	55.6 (20)	
Male	39.4 (138)	44.4 (16)	
Intersex	0.3 (1)	0 (0)	
Gender (% [*n*])			0.802
Male or primarily masculine	38.0 (133)	47.2 (17)	
Female or primarily feminine	60.3 (211)	52.8 (19)	
Neither male nor female	0.6 (2)	0 (0)	
Do not know	0.3 (1)	0 (0)	
Identifies as something else	0.9 (3)	0 (0)	
Ethnicity (% [*n*])			0.734
Black/African American	5.1 (18)	11.1 (4)	
White	77.7 (272)	80.6 (29)	
Latino/White Hispanic	4.3 (15)	0 (0)	
Multiracial	2.9 (10)	0 (0)	
Asian/Asian American (East Asian, Vietnamese, Chinese, Korean, Indian, Filipino)	6.3 (22)	5.6 (2)	
Native Hawaiian	0.3 (1)	0 (0)	
White Native American/Indian	0.9 (3)	0 (0)	
Romanian	0.3 (1)	0 (0)	
DR	2.3 (8)	2.8 (1)	
Place of birth (% [*n*])			0.000
The United States	96 (336)	69.4 (25)	
Canada	0 (0)	25.0 (9)	
Western Europe	1.7 (6)	0 (0)	
Africa	0.3 (1)	0 (0)	
Asia	0.9 (3)	2.8 (1)	
Latin/South America	0.3 (1)	0 (0)	
Other	3 (0.9)	2.8 (1)	
Education (% [*n*])			
Some elementary school	0.3 (1)	0 (0)	0.312
Some high school/secondary school	0.9 (3)	0 (0)	
High school/secondary school degree	7.4 (26)	5.6 (2)	
Some college/university/vocational school	24.3 (85)	16.7 (6)	
College/university/vocational school degree	181 (51.7)	47.2 (17)	
Master's degree/professional school/PhD	15.4 (54)	30.6 (11)	
Income (USD) (% [*n*])			0.509
< $60, 000	55.4 (194)	44.4 (16)	
> $60,000	43.4 (152)	44.4 (16)	
DR	1.1 (4)	11.1 (4)	
Chronic pain conditions (% [*n*])			
Arthritis	34.0 (119)		
Low back pain	60.9 (213)		
Vulvodynia	2.0 (7)		
Chronic pain because of surgery	13.7 (48)		
Inflammatory bowel disease	7.1 (25)		
Fibromyalgia	9.7 (34)		
Other chronic pain condition	23.1 (81)		

Because of missing data, multiple responses, and rounding, not all percentages add up to 100.

DR, decline response.

To assess discriminant validity, participants with chronic pain were compared with individuals with diabetes and no self-reported chronic pain conditions. To be eligible, participants were required to have a 95% hit approval rate on MTurk, be aged at least 18 years, reside in the United States, and self-report diabetes but no chronic pain condition. Thirty-six individuals were included only in the analysis of discriminate validity after screening (see Procedures for further screening methods). Further sociodemographic data are listed in Table 1.

### 2.2. Measures

#### 2.2.1. Chronic Disease Helplessness Survey

Items for the CDHS were first generated based on documented presentations of LH in the animal and human literature (Appendix, available at http://links.lww.com/PR9/A150). Maier and Seligman^19^ argued for a far-reaching set of deficits in response to uncontrollable stress that spans across motivational/motor, cognitive, and emotional symptoms, which they referred to as LH. For this reason, the CDHS was written to capture these 3 domains of helplessness presentation including motivational/motor (“Everything seems to take too much effort.”), cognitive (“I don't believe new treatments will be effective.”), and the emotion (“I get irritated often.”) deficits. Participants responded on a 5-point scale with the anchors: not true, rarely true, somewhat true, mostly true, and very true.

#### 2.2.2. Pain intensity

The Brief Pain Inventory Short-Form is a 15-item scale including 2 multi-item subscales measuring the intensity of physical pain sensations and interference of pain with general daily functioning.^4^ Pain intensity is measured for pain in the last 24 hours at its, current, worst, and average intensity using a numeric scale from 0 to 10, “no pain” to “pain as bad as you can imagine,” respectively. Participants respond to interference questions by indicating the severity that pain interferes with each activity, from “does not interfere” to “interferes completely.” There is much evidence of the reliability and validity of the BPI, including acceptable internal consistency in chronic nonmalignant pain for both intensity items (α = 0.85) and interference items (α = 0.88).^35^

### 2.2.3. Pain helplessness and control

The Pain Catastrophizing Scale is a 13-item scale that asks participants to reflect on past painful experiences and rate the extent to which they felt thoughts and experiences related to rumination, magnification, and helplessness subscales (PCS).^32^ Participants respond on 5-point scales from not at all (0) to all the time (4). Internal consistency has been shown to be acceptable for the helplessness subscale used in this survey (helplessness = 0.78).^32^

The Survey of Pain Attitudes (SOPA) is a 57-item scale assessing pain-related beliefs, including the 10-items subscale of the extent to which patients believe they can control their pain experience.^13^ Participants respond to items on a 5-point scale ranging from very untrue (0) to very true (4). The SOPA has been shown to exhibit acceptable reliability, internal consistency, and criterion validity.^12–14^

#### 2.2.4. General self-efficacy

There is evidence that living with uncontrollable chronic pain leads to a generalized sense that the individual cannot learn techniques to control pain, even experimental pain they have not previously experienced,^21,25,27,37^ or even their environment, as evidenced by poorer construal of goals not related to their pain experience such as work-related goals.^16,17^ For this reason, we predicted that CDHS helplessness would be associated with feeling of low self-efficacy over day-to-day activities not directly related to pain symptoms. The General Self-efficacy Scale (GSE) is a 10-item scale designed to assess optimistic beliefs in one's own ability to cope with difficult demands in life.^28^ Participants respond on a 4-point scale ranging from not at all true (1) to exactly true (4). The GSE has demonstrated acceptable internal reliability between 0.76 and 0.90 (Schwarzer).^28^

#### 2.2.5. Fatigue severity

The Fatigue Severity Scale (FSS) is a 9-item scale of the severity of fatigue during daily activities of patients with various disorders.^18^ Participants respond to scale questions on a scale from strongly disagree (1) to strongly agree (7). The FSS has been previously used in a population with chronic neck pain and exhibited sufficient internal reliability between 0.81 and 0.89.^34^

### 2.3. Procedures

To recruit participants with certain chronic conditions older than 18 years through MTurk, an initial screening survey was used. The screening survey consisted of many distractor questions related to daily functioning, history of brain injury, and internal magnetic resonance imaging contraindications to distract participants from the actual purpose of the screener. The chronic condition question contained a checklist of 33 responses with a range of chronic conditions, including 7 responses pertaining to a chronic pain condition (eg, fibromyalgia, arthritis, chronic pain because of surgery, other chronic pain conditions, etc.) and diabetes. The chronic condition question also contained conditions that are highly unlikely to occur in an adult, North American population, including Kuru, Pica, and Progeria, to identify individuals selecting conditions at random. This question was presented twice during the screening survey with the chronic conditions in a separate randomized order to ensure participants are not simply selecting from the same order of chronic conditions. To be forwarded the CDHS development survey link, participants with chronic pain were required to select chronic pain conditions for both chronic condition questions on the screener, not select the 3 unlikely chronic conditions, and not select more than 6 chronic conditions because most of the participants in the screened population did not report more than 6 chronic conditions, and this cutoff was defined to prevent individuals who selected a large number of conditions to increase their chance of study eligibility. Participants were compensated for completing the screener. Owing to the low response rate by individuals with diabetes to the MTurk screener (N = 24), 12 additional individuals with diabetes were recruited by sharing the screening survey to online Reddit forums for chronic health conditions, Facebook, and Twitter.

The MTurk IDs of eligible participants were input into the CDHS development survey on MTurk, or the survey link was sent to participant emails. Participants were first directed to the letter of information and consent. Completion of the survey took approximately 30 minutes. The chronic condition questionnaire was presented again, and participants were not included in the chronic pain analyses if they did not again indicate a chronic pain condition and similarly for participants with diabetes. An attention check was included that required participants to attentively read question instructions and respond based on the detailed instructions. Participants who failed the attention check were compared with those who passed on questionnaire performance for all analyzed questionnaires, and no significant differences were found. Exploratory factor analysis (EFA) was also performed with and without those who failed, and the factor structure was not significantly altered with their inclusion. For this reason, those who failed the attention check were also included in analyses. Participants with chronic pain and diabetes were then compensated. This study was reviewed by the Queen's University General Research Ethics Board.

### 2.4. Data and statistical considerations

A decline response option was provided for survey responses; therefore, sample sizes varied and will be reported with results. The first 200 eligible participants who responded to the survey were used to perform EFA. Under moderately good conditions seen in this survey (ie, a minimum of 3 variables per factor and communalities between 0.40 and 0.70), 200 participants were sufficient for EFA.^7^ EFA was conducted using IBM SPSS 26 (IBM Corp, Armonk, NY). A maximum likelihood estimation of fit with oblique rotation was used to reduce the number of CDHS variables to allow the factors to correlate. To determine the number of factors to be extracted, a scree plot, parallel analysis (PA), and calculation of the root-mean-square error of approximation (RMSEA) fit index were performed. The parallel analysis was computed using SPSS syntax created by O'Connor.^22^ Root-mean-square error of approximation was conducted using the program FITMOD.^8^

The final 150 eligible participants who completed the survey were included in confirmatory factor analysis (CFA). Confirmatory factor analysis was performed using a maximal likelihood fitting procedure with the Lavaan package in RStudio.^26^ Because there is a little consensus on fit cutoff criteria that indicate good model fit,^3^ the use of multiple fit indices is recommended when evaluating model fit.^9,11^ Three indices of model fit were assessed: RMSEA, standardized root-mean-square residual (SRMSR), and comparative fit index (CFI). Cutoffs consistent with the recommendation made by Hu and Bentler (1998) would include close fit (<0.050), acceptable fit (0.051–0.080), mediocre fit (0.081–0.100), and unacceptable fit (>0.100) for RMSEA, values of less than 0.08 for acceptable SRMSR, and values greater than 0.95 for acceptable CFI.

For assessing convergent and discriminant validity, participant responses from the EFA and CFA were combined (N = 350). Convergent validity was assessed by calculating Pearson correlations between the CDHS total and subscales and the control subscale of the SOPA, helplessness subscale of the PCS, the GSE, and the FSS using SPSS. Discriminant validity was assessed by comparing responses of individuals with chronic pain on the CDHS with individuals with diabetes using independent sample *t* test in SPSS.

## 3. Results

### 3.1. Demographics

*T* tests were used for continuous variables, and χ^2^ analyses were used for categorical variables to examine any demographic differences between those with chronic pain and diabetes (Table 1). The mean age of participants with chronic pain was 41.89 years (*SD* = 13.24). The mean age of participants with diabetes was 42.0 years (*SD* = 15.66). Those with chronic pain significantly differed from those with diabetes only on their place of birth, χ^2^(6, N = 386) = 93.108, *P* < 001. The groups did not significantly differ on age, biological sex, gender, ethnicity, education, or income. Further sociodemographic are listed in Table 1.

### 3.2. Exploratory factor analysis

Before conducting factor analysis, the CDHS was evaluated for factorability. All items were correlated with at least 1 item over *r* = 0.3, and no items were correlated greater than *r* = 0.9.^33^ The Kaiser–Meyer–Olkin measure of sampling adequacy fell within the marvelous range, 0.90.^15^ The Bartlett test of sphericity indicated adequate redundancy, χ^2^(325) = 2932.17, *P* < 0.001.^2^ Assessment of the anti-image matrix were all above the minimum of 0.5.^33^ Based on these results, we concluded that the CDHS has satisfactory factorability.

**Figure 1.**
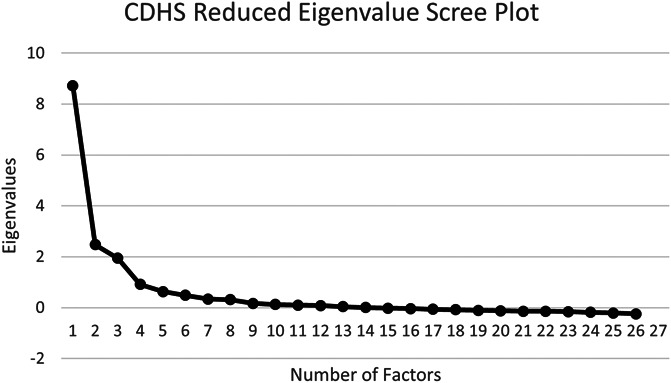
The number of factors obtained was determined using a scree plot of the reduced eigenvalue correlation matrix. CDHS, Chronic Disease Helplessness Survey.

Parallel analysis of reduced eigenvalues suggested a 6-factor structure. Because parallel analysis is known to potentially overfactor, 6 factors were considered the upper limit of factors that could be considered.^6^ Examination of a reduced eigenvalue scree plot revealed a factor structure of 3 factors (Fig. 1). There was not a clear point across models where gains in RMSEA model fit leveled off. However, a 3-factor model was found to have mediocre fit (RMSEA = 0.081, CI 90% [0.071, 0.091]) and a 4-factor model, acceptable fit (RMSEA = 0.067, CI90% [0.055, 0.078]). For this reason, we also examined a 4-factor model of the CDHS in addition to the 3-factor model supported by the scree plot.

Items were then examined for poor loadings of less than |0.40| and cross-loadings on multiple factors. Poor or cross-loading items were iteratively removed to ensure subscales measured only 1 construct,^30^ resulting in a 15-item version of the 4-factor CDHS. Examination of the final 4-factor structure revealed 3 factors that distinctly pulled from the theoretically generated helplessness domain of motivational/motor (1), cognitive (2), and emotional (4) deficits. The third factor seemed to capture feelings of perceived energy to stay motivated but did not fit within the interests of the constructed scale. Although the 4-factor structure exhibited slightly better fit based on the RMSEA than the 3-factor model, the scree plot indicated a 3-factor structure. The conceptual basis of the scale was balanced against the fit indices, and the inclusion of the fourth factor did not result in a theoretically distinct factor, which improved the utility of CDHS. For this reason, the 3-factor structure was retained. Items included in this third factor were removed, and a 3-factor structure was examined, which is within the bounds of reason based on our model fit indices that indicate reasonable fit for the 3-factor model. After removal of poor or cross-loading items, the 3-factor structure consisted of 12 items theoretically consistent with each domain of motivational/motor, cognitive, and emotional deficits (Table 2).

**Table 2:** Three-factor 12-item CDHS loadings.

Factor 1	I seem to sit around watching life go by.	0.889
	Everything seems to take too much effort.	0.844
	I spend a lot of time sitting or lying down.	0.758
	I find it difficult to get up and do things.	0.735
	I feel lethargic.	0.679
	It is hard for me to start new tasks.	0.657
Factor 2	When I hear about a new treatment, I think “why bother?”	0.880
	It is pointless to keep trying new cures.	0.834
	I do not believe new treatments will be effective.	0.622
Factor 3	I feel stressed out.	0.723
	I get irritated often.	0.666
	I am able to face new challenges in a calm manner.	0.625

CDHS, Chronic Disease Helplessness Survey.

### 3.3. Model evaluation

CFA was undertaken for the 3-factor model of the CDHS. Based on the RMSEA, SRMR, and CFI indicators of model fit, the CDHS was found to have reasonable fit. The RMSEA was 0.081, with 90% CI [0.056, 0.104], *P* = 0.022, or mediocre fit. The SRMR and CFI for this sample indicated reasonable fit (SRMR = 0.064, CFI = 0.95). Cronbach alpha values also indicated a high degree of internal consistency for the cognitive (α = 0.812), emotional (α = 0.762), and motivational/motor (α = 0.909) subscales. Based on these results, the 3-factor structure of the CDHS was retained.

### 3.4. Convergent and discriminant validity

To assess convergent validity, Pearson correlations were conducted. As hypothesized, the total score of the CDHS and all 3 subscales were associated with greater current, average, and worst pain intensity on the BPI, pain interference on the BPI, the helplessness subscale of the pain catastrophizing scale, feelings of fatigue, and lower general self-efficacy over one's life (except for the CDHS cognitive subscale) and control over their pain experience (Table 3). Although the CDHS was correlated with measures of convergent and discriminate validity, correlations were not high enough to indicate redundancy with these measures.

**Table 3:** Convergent validity of the Chronic Disease Helplessness Survey (CDHS) scale.

	CDHS total (*r*(*N*))	Motivational deficits (*r*(*N*))	Cognitive deficits (*r*(*N*))	Emotional deficits (*r*(*N*))
BPI pain intensity-current	0.377** (349)	0.354** (348)	0.327** (349)	0.233** (349)
BPI pain intensity-average	0.349** (349)	0.306** (348)	0.281** (349)	0.217** (349)
BPI pain intensity-worst	0.306** (349)	0.272** (348)	0.200** (349)	0.218** (349)
BPI pain interference	0.604** (349)	0.577** (348)	0.307** (349)	0.469** (349)
Fatigue severity	0.621** (347)	0.641** (346)	0.234** (347)	0.485** (347)
PCS helplessness	0.620** (347)	0.561** (346)	0.377** (347)	0.494** (347)
General self-efficacy	-0.386** (347)	-0.377** (346)	-0.077 (347)	-0.463** (347)
SOPA control	-0.492** (348)	0.456** (347)	-0.355** (348)	-0.304** (348)

*P < 0.05 (2-tailed); **P < 0.01 (2-tailed).

BPI, Brief Pain Inventory; PCS, Pain Catastrophizing Scale; SOPA, Survey of Pain Attitudes.

**Figure 2.**
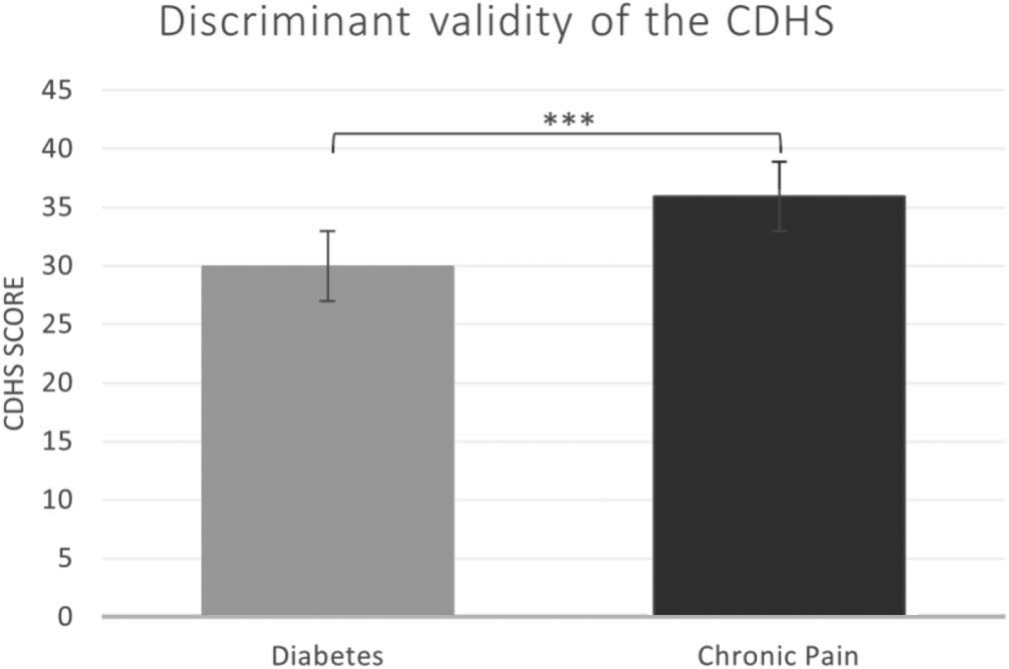
Discriminant validity was assessed by comparing individuals with chronic pain with those with diabetes using independent sample *t* test. CDHS, Chronic Disease Helplessness Survey.

Discriminant validity was assessed by comparing individuals with chronic pain with those with diabetes using independent sample *t* Test. Individuals with diabetes (*M* = 29.97; *SD* = 10.47) displayed significantly lower CDHS scores in comparison with individuals with chronic pain (*M* = 35.92; *SD* = 9.82), *t*(384) = 3.44, *P* = 0.001; *d* = 0.59 (Fig. 2).

## 4. Discussion

There are many scales that capture various control constructs, and, particularly in the area of pain measurement, multiple scales assess perception of controllability over the pain experience. However, the construct of LH is not defined by the lack of control over an aversive stimuli alone.^19^ Helplessness results in far-reaching deficits across motivational/motor, cognitive, and emotional domains.^19^ This study has developed a three-factor scale intended to capture each of these LH effects. Based on the performed CFA, the three-factor structure has reasonable fit and high internal consistency.

Based on all the assessed measures, the CDHS demonstrated optimal construct validity for its intended purpose. Most of the correlations fell between small to moderate correlations, and the largest observed correlation was 0.641, suggesting that it is not redundant with other measures of control constructs, including the PCS, SOPA, and GSE. The PCS subscale for helplessness and the control subscale of the SOPA were expected to have at least a moderate correlation with the CDHS because all 3 related to stressor controllability. Unsurprisingly, both were correlated, and the PCS scale demonstrated the greatest association; however, the CDHS and its subscales were not entirely redundant with this measure, suggesting that the domains of LH deficits tap into variance in the experience of helplessness not captured by the subscale of the PCS. This divergence is expected because LH is the common but not inevitable cognitive and behavioural state created by long-term exposure to uncontrollable stressors. LH is trans-situational in that the evident behaviors occur in an environment that the organism views as being distinct from that in which the original stressor occurred.^20^ In other words, helplessness would result in perceptions of low control over their life at large, not only the pain condition. As predicted, CDHS helplessness was significantly negatively correlated with general self-efficacy.

It was hypothesized that individuals high in helplessness related to their pain condition would exhibit greater pain severity and interference because the stressor would theoretically be perceived as more aversive and uncontrollable. As expected, pain intensity and pain interference scores were correlated with feelings of helplessness. The construct validity of the CDHS is supported by the correlation with pain intensity. However, pain interference may be an even greater indicator conceptually because although pain intensity and interference are highly correlated, it is possible for participants with high pain intensity to also demonstrate adaptive coping strategies and low feelings of helplessness over their pain. Greater interference may be a more accurate indicator of poor pain control and ability to learn pain-related escape strategies.

Helplessness manifests behaviorally through motivational and motor deficits such as escape-related responses,^1,10,23,29,39^, decreased aggressive and competitive behavior,^5,24^ and reductions in spontaneous motor behavior.^38^ As predicted, the CDHS and, particularly, the motivation/motor subscale of the CDHS were associated with the FSS. In fact, all subscales were associated with the FSS, with the motivation/motor subscale being the highest correlation, as would be expected. The construct validity of the CDHS and, particularly, the motivation/motor deficit subscale of the CDHS demonstrated sufficient validity.

Pain disorders are frequently treatment resistant, leaving those experiencing the disorders frustrated and despondent. It has been shown that living with uncontrollable chronic pain results in a generalized deficit in learning new techniques to control pain, including experimental pain they have not previously experienced^21,25,27,37^ or even their environment.^16,17^ Exposure to uncontrollable stress results in a reduced ability to learn about contingent relationships between a stimulus and an avoidant response. This inability to learn new strategies to cope with symptomology may result in poor response to treatments and could potentially be a critical determinant of suffering in chronic conditions. Identification of those experiencing learned helplessness and targeting their reduced ability to learn about contingent relationships between stimulus and an avoidant response might be a mechanism for change in outcomes after psychological treatments, such as cognitive behavioral therapy. None of the extant helplessness measures to date assess LH-related deficits for identifying and mastering control strategies, as outlined by Maier and Seligman, but instead only focus on perceptions of control. The CDHS provides a psychometrically sound measure of these LH-related deficits.

### 4.1. Limitations and future research directions

This scale was developed so that it may be administered to any population with a chronic condition and questions do not pertain specifically to chronic pain or any other chronic condition. Further validation and factor structure assessment should be performed in the future in other populations with chronic disease. In addition, the domains of LH assessed in this scale were not validated with behavioral paradigms or observations. Future research should seek to manipulate or observe LH behaviors and examine possible associations with the CDHS scale to assess its ecological validity.

The sample and recruitment methods used for this study also introduce limitations to generalizing the results. Participants were primarily recruited using MTurk, which requires access to a computer, Internet, and technological literacy that biases the sample. In this study, recruitment of MTurk has resulted in the underrepresentation of minority populations. Future research should seek to replicate these results in a more ethnically diverse clinical population. In addition, due to difficulty in recruiting individuals with diabetes, an examination of divergent validity and the generalization of results for those living with diabetes are limited by the small sample size of the group with diabetes. However, an examination of group demographics indicates that the clinical populations are relatively similar for the purposes of comparison.

## 5. Conclusion

This study provides preliminary evidence of the psychometric soundness and conceptual validity of the CDHS for measuring the motivational/motor, cognitive, and emotional deficits that occur as a result of LH. Further research is needed to assess the predictive validity of the CDHS by assessing its ability to predict long-term symptomology outcomes for chronic pain conditions and to validate the CDHS in other chronic health conditions. The CDHS provides utility as a self-report measure of LH, classically described by behavioral observations in humans and animals, which does not blur the conceptualizations of other control constructs.

## Disclosures

The authors have no conflicts of interest to declare.

## Appendix A. Supplemental digital content

Supplemental digital content associated with this article can be found online at http://links.lww.com/PR9/A150.
